# Detecting and Monitoring Periprosthetic Joint Infection by Using Electrical Bioimpedance Spectroscopy: A Preliminary Case Study

**DOI:** 10.3390/diagnostics12071680

**Published:** 2022-07-10

**Authors:** Marco Balato, Carlo Petrarca, Pasquale Arpaia, Nicola Moccaldi, Francesca Mancino, Giusy Carleo, Simone Minucci, Massimo Mariconda, Giovanni Balato

**Affiliations:** 1 Department of Electrical Engineering and Information Technologies (DIETI), University of Naples “Federico II”, 80125 Napoli, Italy; carlo.petrarca@unina.it (C.P.); pasquale.arpaia@unina.it (P.A.); nicola.moccaldi@unina.it (N.M.); francesca.mancino@unina.it (F.M.); 2 Interdepartmental Research Center on Management and Innovation in Healthcare, (CIRMIS), University of Naples “Federico II”, 80131 Napoli, Italy; massimo.mariconda@unina.it; 3 Department of Neuroscience and Reproductive and Odontostomatological Sciences, University of Naples “Federico II”, 80131 Napoli, Italy; giusy.carleo@unina.it; 4 Department of Economics, Engineering, Society and Business Organization (DEIM), University of Tuscia, 01100 Viterbo, Italy; simone.minucci@unitus.it; 5 Department of Public Health, University of Naples “Federico II”, 80131 Napoli, Italy; giovanni.balato@unina.it

**Keywords:** biofilm infections, diagnostic methods, electrical bioimpedance spectroscopy, diseases diagnostic, prediction

## Abstract

A method to detect the presence of infection after Total Joint Arthroplasty is presented. The method is based on Electrical Bioimpedance Spectroscopy and guarantees low latency, non-invasiveness, and cheapness with respect to the state of art. Experimental measurements were carried out on a singular patient who had already undergone bilateral Total Knee Arthroplasty. He was affected by a hematogenous Periprosthetic Joint Infections on the left knee. The right knee was adopted as the reference. Measurements were acquired once before the surgical procedure (Diagnosis Phase) and twice in the postoperative phases (Monitoring Phase). The most relevant frequency range, for diagnosis and monitoring phases, was found to be between 10 kHz to 50 kHz. The healing trend predicted by the decrease of impedance magnitude spectrum was reflected in clinical and laboratory results. In addition, one month after the last acquisition (two months after the surgery), the patient fully recovered, confirming the prediction of the Electrical Bioimpedance Spectroscopy technique.

## 1. Introduction

Biofilm-related Infections (BIs) are extremely serious health problems due to the low success rate of treatment procedures, correlated to the presence of extracellular biofilm matrix and the bacterial metabolic state [1,2]. In orthopedic surgery, prosthetic joints and osteosynthesis devices (i.e., intramedullary nails, plates, screws, wires, etc.) represent the ideal setting for biofilm development [3]. BIs involving orthopedic implants, such as Periprosthetic Joint Infections (PJIs), represent one of the most devastating complications that occur in 0.5% to 2% of joint replacements and over 15% of open fractures [4,5], and their incidence is expected to increase over time [6]. In fact, smoke and alcohol abuse, diabetes mellitus, avascular necrosis of femoral head, rheumatoid arthritis, cardiovascular disease, and osteoarthritis are known to be the major patient-related risk factors responsible of increasing PJIs’ occurrence rate [7]. Regardless of the number of cases affected by septic complications, treating these infections is highly demanding and can represent a substantial burden for the healthcare system [6]. In particular, the treatment consists of the mechanical removal of all infected tissues (debridement), contaminated implant, and prosthesis in one or two surgical stages associated with long-lasting antibiotic therapy. Two-stage revision arthroplasty is a well-established method of care for patients with chronic PJIs and provides the use of a cement spacer and, afterwards, the implantation of a revision prosthesis once the infection is considered eradicated [8,9,10]. Although the two-stage technique guarantees a high success rate, it typically results in an increased risk for blood loss, iatrogenic bone defects, long hospitalization time, and, consequently, patients’ high disability. The first step to PJIs’ successful management is the accurate identification of the septic process, thus reducing the risk of infection recurrence estimated to range between 7% to 17% [11]. Currently available procedures and/or techniques to detect the PJIs are time-consuming and often ineffective. In many cases, the results are difficult to evaluate and not available in the desired time scale for taking quick corrective measures and, therefore, fail to guide the clinician/technician in identifying contaminated sites. Among such techniques, quantitative cultures remain the gold standard but have a long Turnaround Time; moreover, the bacteria incorporated in the biofilm can be released as very few cells from the samples causing false negatives or complicating the interpretation in case of detection of skin commensals. Chemical detachment of bacteria or sonication of orthopedic devices improved the sensitivity of the culture but did not solve the problem of false negatives [12]. The application of molecular methods on the sonication liquid to identify the etiological agent cannot, for now, replace traditional diagnostics [13]. Therefore, an accurate, reliable, non-invasive, and rapid test for the diagnosis of PJIs is not yet available. The above discussion highlights the necessity of effective management of PJIs including detection and real-time monitoring phases. Recent studies suggest the enforcement of Electrical Bioimpedance Spectroscopy (EBS) as a non-invasive, portable, and low-cost tool for diagnosis and monitoring of BIs [14,15]. The development of this technique is increasing due to the inaccessible nature of the environments in which the biofilms grow. In addition, EBS seems to be a promising solution to evaluate: (i) fracture fixation [16]; (ii) the pressure-induced tissue damage in case of pressure ulcers [17,18]; (iii) the differences in contracted state, cellular metabolic activity, and extracellular fluid between healthy subjects and subjects who had suffered muscle injury [19]; (iv) the variation in soft-tissue hydration and in cell membrane integrity [20]; (v) the effect of excessive thickening of tissue between the prosthesis and the bone in low-depth prostheses [21]. Despite the practical advantage and established measurement properties of EBS, its involvement in effective management of PJIs is not yet clear. The present paper aims to investigate the EBS approach’s role in diagnosing PJIs and monitoring the effectiveness of the treatment choice. We describe a singular case report of a patient who underwent bilateral Total Knee Arthroplasty (TKA) in 2012 and 2013. Moreover, in February 2022, the patient developed a hematogenous PJI on the left knee (unhealthy knee) which underwent an EBS investigation. In addition, the right knee (healthy knee) was considered the reference. The measurement campaign, carried out by a suitable test apparatus, was conducted in two phases: once before the surgical procedure (Diagnosis Phase) and twice in the postoperative phases (Monitoring Phase). The EBS experimental results were compared with obtained clinical and laboratory analyses.

## 2. Materials and Methods

The patient had to sign the informed consent prior to the tests and all procedures were carried out in compliance with Helsinki guidelines. Tests were conducted in an air-controlled temperature room of Policlinico, University Federico II, Naples.

### 2.1. Case Study

**Figure 1:**
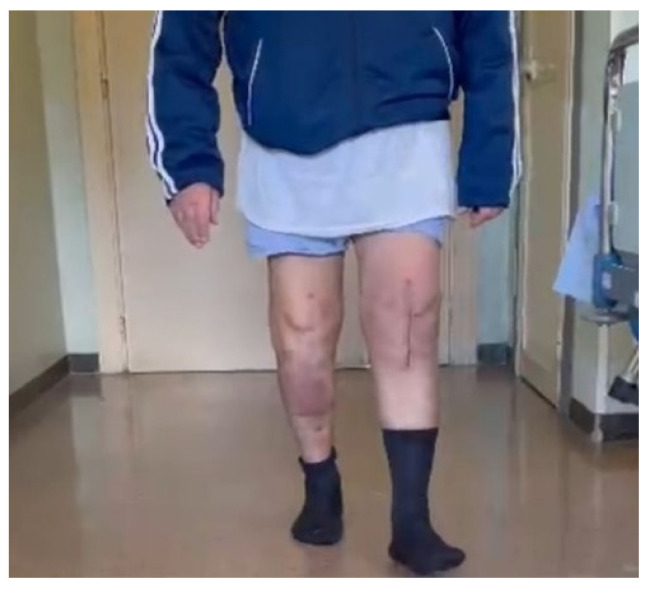
Patient’s condition two months after surgery.

A 79-year-old man with a 10-year history of atrial fibrillation and arterial hypertension underwent TKA in September 2012 and left TKA in December 2013. The postoperative period was uneventful until September 2021. The patient was admitted to another hospital because of fever with shaking chill, increasing movement limitation, and swelling in the left knee, treated with empirical antibiotic therapy for two weeks. Our observation in December 2021 revealed extensive erythema and swelling at the surgical site and reduced range of motion with a knee extension deficit of 30 degrees. The patient was apyretic, but laboratory examination revealed an Erythrocyte Sedimentation Rate (ESR) of 87 mm/h and a C- Reactive Protein (CRP) level of 29 mg/L (normal < 5). Leukocyte and hemoglobin counts were normal. Knee joint aspiration was performed for laboratory and microbiological investigations, which showed an elevated WBC count (117.376 cells/μL) with high polymorphonuclear cells (91%) levels. In February 2022, the patient underwent a surgical procedure that consisted of accurate debridement, knee prosthesis removal (left knee), and an implant of a mobile spacer (a two-stage technique). We decided to implant a “metal on poly” mobile spacer, which consisted of a femoral prosthesis in titanium (cruciate retaining femoral component) and an ultra-congruent insert in polyethylene as described by Hofmann et al. in 1995 [22]. We administered teicoplanin (8 mg/kg body weight) based on our hospital protocol while awaiting culture results to ensure adequate coverage for methicillin-resistant Staphylococcus aureus. Cultures of intraoperative tissue samples yielded methicillin-susceptible Staphylococcus aureus. Intravenous antibiotic therapy was continued for two weeks without significant adverse effects, based on the outpatient parenteral antibiotic therapy (OPAT) model. Two weeks after the surgical procedure, intravenous antibiotic therapy was switched to oral treatment with Trimethoprim/Sulfamethoxazole 800/160 mg (thrice daily) and minocycline (100 mg twice a daily) for eight weeks. Two months after the surgery, the patient has fully recovered, as shown in Figure 1, for all motor functions. In addition to the standard PJIs’ management strategies, the patient underwent EBS procedures to verify their abilities in providing PJIs’ diagnostic and real-time monitoring functions.

### 2.2. Measurement Instrumentation

**Figure 2:**
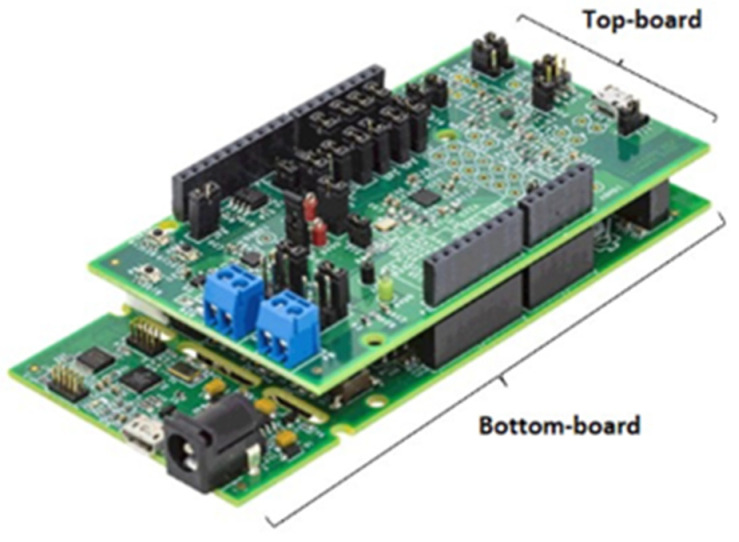
AD5940 Evaluation Boards for 4-wire bioimpedance spectroscopy.

The measurement instrumentation used in the experiments was based on the microchip AD5940 by Analog Devices [23]. Indeed, the feasibility study was aimed at laying the groundwork for prototyping a wearable solution that can be integrated into a knee brace. Analog Devices provides two boards, namely EVAL-AD5940BIOZ (Top-board) and EVAL-ADICUP3029 (Bottom-board) to develop a custom firmware and test the microchip (Figure 2). The system applies a voltage settled by the operator (potentiometric configuration) in the range (10–750) mV. The maximum peak current is kept below IEC 60601 limits [24] by means of appropriate resistors and capacitors along the amperometric line. The EVAL-AD5940BIOZ is an Arduino platform, which hosts a microcontroller ultra-low power Arm Cortex-M3 to perform bio-electric measurements [23]. A 16-bit ADC with both 800 kSa/s and 1.6 MSa/s options and a voltage 12-bit DAC with output range of 0.2 V to 2.4 V and 200 kSa/s output are provided [24]. The bottom-board includes an integrated mixed-signal micro-controller systems, namely an ADuCM 3029 chip, which processes signals received by the up-board. Custom firmware was developed in the IAR Embedded Workbench Integrated Development Environment, and a user interface was realized in C-Sharp to allow the control of the bioimpedance meter. The instrument is connected to the PC through a USB port for power supply and data transmission. Bio-impedance measurements are implemented by means of a 4-wire configuration. A Digital Fourier Transform is performed by the AD5940 on the current and voltage measurements, respectively. Then the microcontroller uses the real and imaginary DFT results for current and voltage at specific frequency to calculate the unknown impedance.

### 2.3. Setup

**Figure 3:**
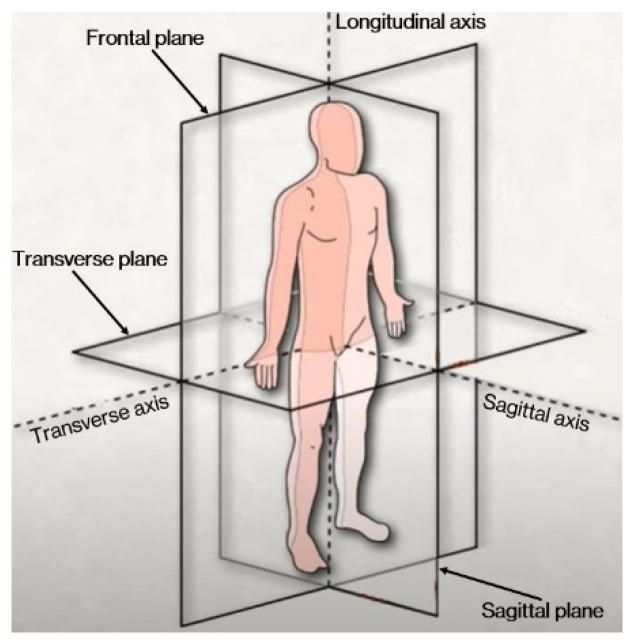
Reference Cartesian System used for electrodes positioning.

During the measurements, the impedance meter was connected to a laptop (set to battery power mode) for signal acquisition and all electronic devices were removed from the measurement zone. Before acquisitions, knee circumference, patella length and leg thickness above and below the patella were measured to exclude macroscopic asymmetries between the two knees, as reported in Table 1. The patient was subjected to three measurement cycles; namely: (i) before surgery, (ii) one week after surgery and (iii) one month after surgery. For each cycle, three measurements were realized at 100 frequencies evenly spaced from 100 Hz to 100 kHz on a logarithmic scale. Four FIAB 500 electrodes of 14 mm × 36 mm were placed on the knees as shown in the following. After the electrode positioning, the patient was asked to sit for the entire duration of the experiment. Tetrapolar impedance measurements are affected by peculiar uncertainty sources [25]. Nevertheless, the minimization of the impact of skin-electrode interface on the impedance measurement was assumed as the main goal to pursue. Indeed, by means of the tetrapolar setup, the voltage drop is only due to the current circulating subcutaneously. To make the experiment reproducible, a system of cartesian axes (transverse, sagittal, longitudinal) was defined as shown in Figure 3 and three planes were identified: sagittal, frontal and transverse. The position of the electrodes was identified through the choice of appropriate points in the defined space.

**Figure 4:**
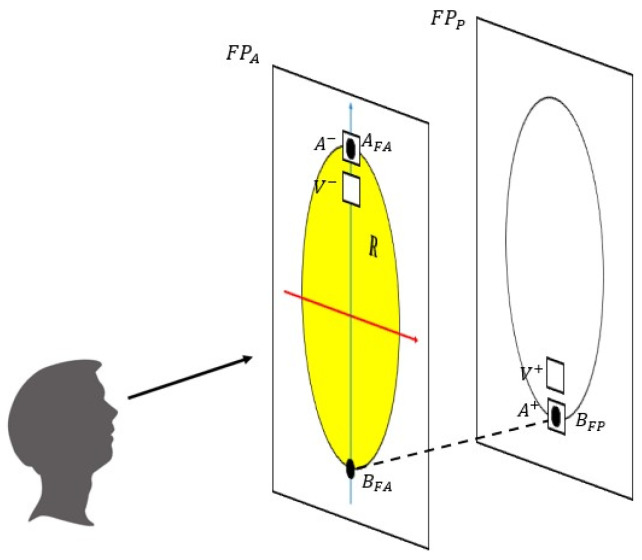
Procedure of electrodes position identification. In yellow, the projection of the patella in the anterior frontal plane. A_FA_ and B_FP_ are identified placement points. A^−^ and A^+^ are the ampero-metric electrodes, V^−^ and V^+^ are the volt-metric electrodes.

**Figure 5:**
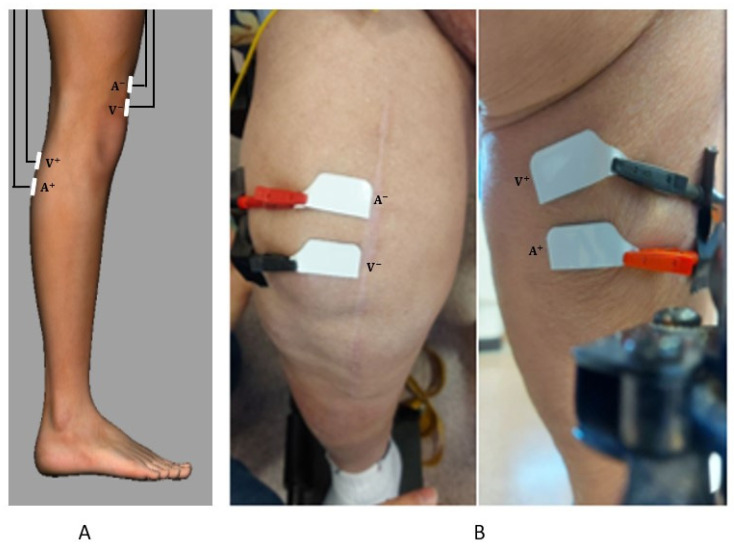
(**A**): Sagittal view of the leg model with electrode placement (**B**): actual electrode placement on the patient.

Two frontal planes (anterior and posterior) are defined in Figure 4. The planes identify the two planes tangent to the anterior and posterior surfaces of the knee. In Figure 4, the surface R is defined as the projection of the patella in the anterior frontal plane. The points A_FA_ and B_FA_ are identified as the intersection of the longitudinal axis with the R surface contour; the point B_FP_ is identified as the orthogonal projection of the point B_FA_ in the frontal posterior plane. Rear-front configuration (Figure 5A): A^−^ is centered in A_FA_ with the longer side parallel to the transverse axis and V^−^ is placed 1 cm below. A^+^ is centered in B_FP_ and V^+^ is placed 1 cm above. The measurements were realized for both the knees to compare the impedance variation from knee with healthy and infected prosthesis.

The distance between the two impedance spectra is assessed in terms of relative difference, evaluated as follows:

(1) $$\varepsilon_{Z}=∥\frac{Z_{U}\left(\omega_{k}\right)-Z_{H}\left(\omega_{k}\right)}{Z_{H}\left(\omega_{k}\right)}∥$$

where *ω_k_* is the angular frequency used in the experimental measurements and the subscripts *U* and *H* refer to the unhealthy and healthy knee, respectively.

## 3. Results

**Figure 6:**
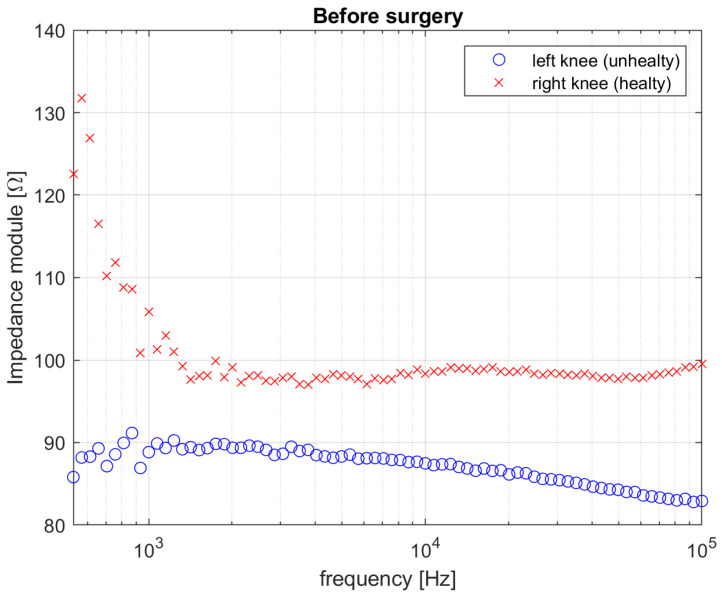
Impedance magnitude value for both right (healthy) and left (unhealthy) knee before surgery.

**Figure 7:**
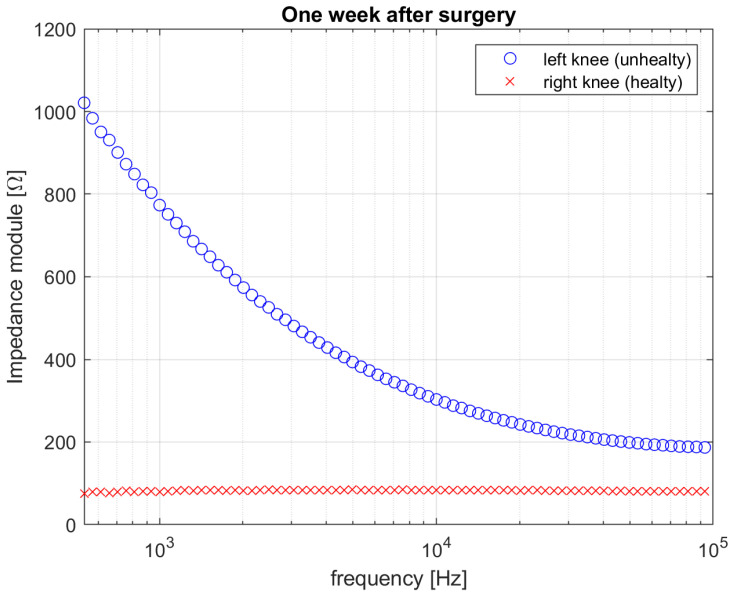
Impedance magnitude value for both right (healthy) and left (unhealthy) knee one week after surgery.

Figure 6 and Figure 7 show the results in terms of comparison between measured impedance magnitude in the rear–front electrodes configuration for both the two knees, acquired before the surgery, one week after the surgery and one month after the surgery, respectively.

The diagrams reported in Figure 6 highlight the global effect of the biofilm presence in the left (unhealthy) knee on the measured impedance. This effect may be attributed to their extracellular fluid structure that exhibits higher real and imaginary parts of the complex dielectric function, resulting in a globally lower impedance spectrum, w.r.t. to the right (healthy) knee. This is justified by the relative distance between the two spectra, which is equal to ε_Z_pre_ = 0.62. 

**Figure 8:**
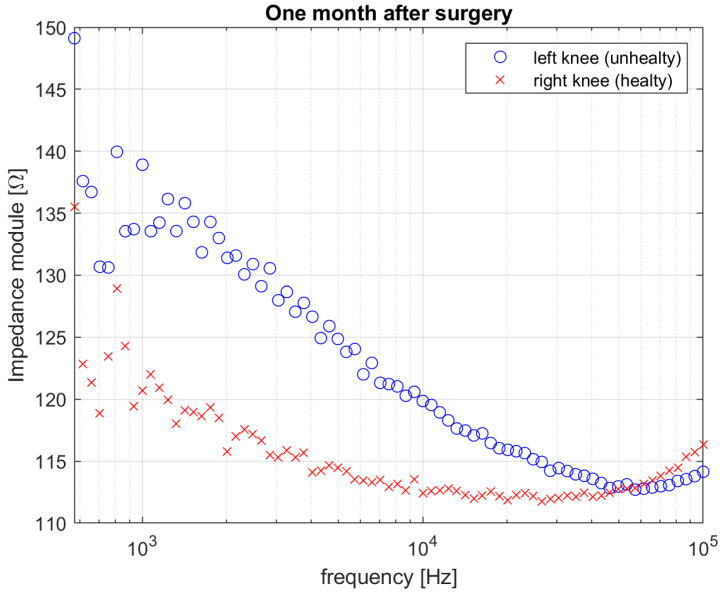
Impedance magnitude value for both right (healthy) and left (unhealthy) knee one month after surgery.

**Figure 9:**
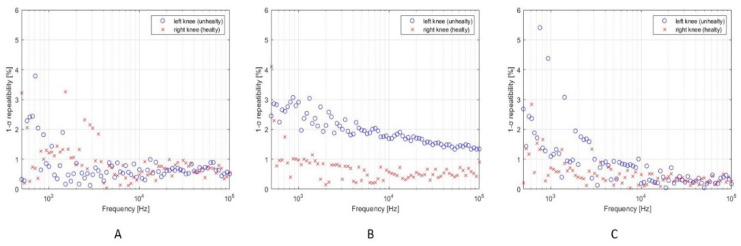
Impedance magnitude 1-σ repeatability values for both right (healthy) and left (unhealthy) knee before surgery (**A**), one week after surgery (**B**), and one month after surgery (**C**).

The impedance spectrum of the unhealthy knee dramatically changes soon after the surgery treatment, as shown in Figure 7. Such a dramatic variation may not only be attributed to the presence of biofilm but more likely to surgical traumatic effects such as debridement, implant of spacer, fluid distribution, tissues removal, hematoma, etc. [26,27]. This is reflected in the measured higher impedance magnitude spectrum w.r.t. the pre-surgery measurement and the right knee measurement shown in Figure 7, and in the relative distance between the two spectra, which is equal to ε_Z_1-week-post_ = 9.55. Finally, the electrical behavior of both of the two knees is reported in Figure 8, in terms of measured impedance magnitude spectrum, one month after the surgery. In this period, the spacer gained its situ and the knee tissues do not experience the traumatic effects of surgery any longer. The impedance magnitude spectrum measurement is compatible with that measured for the healthy knee and is in accordance with the healing trend shown by the patient and confirmed by clinical results. The above is justified also by the relative distance between the two spectra, which is now equal to ε_Z_1-month-post_ = 0.18. The lower distance value, calculated one month after the surgery, highlights that the left knee overall situation is closer to that of the right knee, thanks to the healing process, which is still undergoing and is not completed yet. In fact, it is estimated that a period lasting not less than 3 months is necessary for a full recovery after undergoing TKA. However, it should be noted that even when the healing process can be considered as completed, a certain distance has to be accounted for between the two knees, because of the physiological asymmetry between the left-hand side and the right-hand side of the human body. Future experimental campaigns will also be devoted to identifying a threshold to detect the many stages of the knee healing. Percentage 1-σ repeatability was calculated for each frequency as the percentage ratio between the standard deviation and the mean impedance magnitude. The results are shown in Figure 9. The mean percentage 1-σ repeatability of impedance magnitude were 0.81%, 1.32%, and 0.70% for before surgery, one week after surgery, and one month after surgery measurements, respectively. The repeatability was almost comparable among the three sessions. 

## 4. Discussion

The diagnosis of PJIs represents a challenge for an orthopedic surgeon. In the last few years, different guidelines and diagnostic criteria have been proposed to identify and treat patients affected by PJI. Unfortunately, no ‘gold standard’ exists, and no single test with 100% diagnostic accuracy to detect infection is available. The lack of identification of PJI is related to biofilm, a tridimensional structure of bacteria that prevents the possibility of microbiological analysis in detecting the infective organism. Recently, the attention of physicians has been focused on the concentration of synovial fluid biomarkers in response to bacterial pathogens. Indeed, all the tests, except for microbiological analysis, useful in the diagnostic algorithm of PJI are indirect, therefore evaluating the host response to an infective organism instead of identification of the infection. Alternatively, recent studies suggest the enforcement of EBS analysis as a non-invasive, portable, and low-cost tool able to exceed the limits of current BIs diagnostic and monitoring procedures that are time-consuming and often ineffective, exposing patients to too late or incorrect diagnoses. The preliminary experimental results, as shown in Figure 4, Figure 5 and Figure 6, fully confirm the above statement also for the diagnosis and real-time monitoring of PJIs in a patient with TKA on both sites. In particular, they highlight the capability of EBS technique to discriminate the presence of infection (diagnosis function), the treatment effects and the healing stage (monitoring function). The above is manifested through variation of the impedance magnitude spectrum of the unhealthy knee (blue curve) with respect to healthy knees (red curve). The most relevant frequency range, for diagnosis and monitoring phases, is between 1 kHz to 10 kHz where substantial differences have been found. Finally, the healing trend predicted by the decrease of impedance magnitude spectrum was reflected in clinical and laboratory results. In addition, one month after the last EBS acquisition (two months after the surgery), the patient fully recovered, as shown in Figure 1, in all motor functions, confirming the prediction of the EBS technique. In conclusion, EBS represents a promising solution for accurate management of PJIs, especially for its short turnaround time that lasts only a few minutes. Further work is in progress with the aim of confirming the obtained results on a wider range of cases. In addition, an improved version of the test apparatus is under development to measure, in the optimal frequency range, not only f0rthe impedance amplitude, but also for its real and imaginary parts. The objective is to overcome the significant limitations of our case report study that include (i) lack of ability to generalize, (ii) the inability to establish an accurate cause–effect relationship, and (iii) the danger of over-interpretation of a single case or even a case series (the so-called anecdotal fallacy).

## Figures and Tables

**Table 1:** Parameters considered to ensure homogeneity of the setup.

Body Part	Right Knee	Left Knee
Knee circumference	48 cm	48 cm
Patella length	18 cm	17 cm
Leg thickness above patella	8 cm	10 cm
Leg thickness below patella	8 cm	10 cm

## Data Availability

Not applicable.
